# Epstein-Barr Virus and Human Papillomavirus Infections and Genotype Distribution in Head and Neck Cancers

**DOI:** 10.1371/journal.pone.0113702

**Published:** 2014-11-18

**Authors:** Zeyi Deng, Takayuki Uehara, Hiroyuki Maeda, Masahiro Hasegawa, Sen Matayoshi, Asanori Kiyuna, Shinya Agena, Xiaoli Pan, Chunlin Zhang, Yukashi Yamashita, Minqiang Xie, Mikio Suzuki

**Affiliations:** 1 Department of Otorhinolaryngology, Head and Neck Surgery, Zhujiang Hospital, Southern Medical University, Guangzhou, China; 2 Department of Otorhinolaryngology, Head and Neck Surgery, Graduate School of Medicine, University of the Ryukyus, Okinawa, Japan; The Ohio State University, United States of America

## Abstract

**Objective:**

To investigate the prevalence, genotypes, and prognostic values of Epstein-Barr virus (EBV) and human papillomavirus (HPV) infections in Japanese patients with different types of head and neck cancer (HNC).

**Methods and Materials:**

HPV and EBV DNA, EBV genotypes and *LMP-1* variants, and HPV mRNA expression were detected by PCR from fresh-frozen HNC samples. HPV genotypes were determined by direct sequencing, and EBV encoded RNA (*EBER*) was examined by in situ hybridization.

**Results:**

Of the 209 HNC patients, 63 (30.1%) had HPV infection, and HPV-16 was the most common subtype (86.9%). HPV *E6/E7* mRNA expression was found in 23 of 60 (38.3%) HPV DNA-positive cases detected. The site of highest prevalence of HPV was the oropharynx (45.9%). Among 146 (69.9%) HNCs in which EBV DNA was identified, 107 (73.3%) and 27 (18.5%) contained types A and B, respectively, and 124 (84.9%) showed the existence of *del-LMP-1*. However, only 13 (6.2%) HNCs were positive for *EBER*, 12 (92.3%) of which derived from the nasopharynx. Co-infection of HPV and *EBER* was found in only 1.0% of HNCs and 10.0% of NPCs. Kaplan-Meier survival analysis showed significantly better disease-specific and overall survival in the HPV DNA+/mRNA+ oropharyngeal squamous cell carcinoma (OPC) patients than in the other OPC patients (*P* = 0.027 and 0.017, respectively). Multivariate analysis showed that stage T1–3 (*P* = 0.002) and HPV mRNA-positive status (*P* = 0.061) independently predicted better disease-specific survival. No significant difference in disease-specific survival was found between the *EBER*-positive and -negative NPC patients (*P* = 0.155).

**Conclusions:**

Our findings indicate that co-infection with HPV and EBV is rare in HNC. Oropharyngeal SCC with active HPV infection was related to a highly favorable outcome, while EBV status was not prognostic in the NPC cohort.

## Introduction

Head and neck cancer (HNC) is the sixth leading cancer in the world, with more than 600,000 new cases reported each year [1]. Excessive alcohol and tobacco consumption are two traditional and major risk factors for HNC. In the last two decades, the incidence of HNC, such as carcinoma of the larynx or hypopharynx, has decreased significantly due to preventive strategies targeting these risk factors. However, the incidence of oropharyngeal squamous cell carcinoma (OPC) has increased sharply, particularly in young-onset HNC in the absence of these traditional risk factors, which suggests the involvement of other factors. Human papillomavirus (HPV) is now also an established etiologic factor for the occurrence and development of HNC, especially OPC. An increase in the proportion of HPV-related oropharyngeal cancer was observed in the United States from 1973 to 2004 [2]. Similarly, two retrospective cohort studies from Stockholm, Sweden, showed increases in the proportion of HPV-positive tonsillar cancer from 23.3% in the 1970s to 93% for the period 2006–2007 and in that of HPV-positive base of tongue cancer from 58% for 1998–2001 to 84% for 2006–2007 [3], [4]. In addition, the fact that HPV is detected in non-oropharyngeal HNC suggests that its role in the development and progression of HNC is not limited to the oropharynx [5], but includes the nasopharynx, where Epstein-Barr virus (EBV) is consistently detected in cancer cells from regions of high and low incidence [6].

EBV is an ubiquitous herpes virus that affects more than 90% of the world's adult population and is associated with variety of malignant disorders including Hodgkin's disease, Burkitt's lymphoma, B-cell lymphoma [7], [8], [9], and gastric carcinoma [10]. In the head and neck, the establishment of a latent transforming EBV infection and the potential viral genetic changes that occur in epithelial cells may contribute to the development, growth, and invasive capabilities of nasopharyngeal carcinoma (NPC) [6]. Plasma EBV DNA analysis has proven useful in detecting early NPC in individuals when there is no clinical suspicion of tumor [11]. Several viral proteins of EBV include the latency-associated proteins, latent membrane proteins-1 and -2 (*LMP-1* and *LMP-2*), and the EBV nuclear antigens (*EBNA1–6*) [9], [12]. EBV is classified as type A or B based on linked polymorphism in *EBNA*-*2*, *-3A*, *-3B*, and *-3C* [13]. *LMP-1* has been found to be an EBV oncogene [14]. It comprises a short amino acid cytoplasmic N-terminus (amino acids 1–23), six transmembrane-spanning domains (amino acids 24–186), and a C-terminus (amino acids 187–386). The C-terminus and transmembrane-spanning domains of *LMP-1* are required for the maximal activation of nuclear factor-kappa B (NF-kB), the activation of which is linked to the inhibition of apoptosis [15], [16], [17]. A deletion of 30 base pair (bp) at the 3′C-terminal region of the *LMP-1* gene (*del-LMP-1*) has been reported in NPC in Chinese patients. This deletion can result in loss of amino acids 346–355 in the *LMP-1* protein. The deletion protein has a longer half-life and confers enhanced NF-kappa B and JNK/AP1 signaling activity in epithelial cells. These properties of *del-LMP-1* variant are localized to the transmembrane-spanning domains and may contribute to a more malignant phenotype of NPC [18]. Previous studies have found *del-LMP-1* to be present in more than 75% of NPC patients in Southeast Asia and North Africa [19], [20], [21]. The predominance of specific *LMP-1* variants in NPC may reflect differences in the biological or molecular properties of *LMP-1* variants. Moreover, *del-LMP-1* has lower immunogenicity than the non-del variant, which may give rise to tumor development in immunocompetent hosts via escaping immunosurveillance [22]. However, the prevalence and role of EBV in HNC at sites other than the nasopharynx are controversial. While EBV DNA has been identified by polymerase chain reaction (PCR) in 72–92% of oral SCC, laryngeal SCC, and pharyngeal SCC cases [23], [24], [25], [26], the prevalence of EBV infection based on in situ hybridization (ISH) targeting EBV encoded RNA (*EBER*), the gold-standard assay for determining whether a biopsied tumor is EBV-related, is significantly inconsistent in these cancers [23], [27], [28], [29]. Furthermore, NPC incidence differs among geographic regions and, despite EBV infection occurring extensively worldwide, this indicates that a subtype of EBV is involved in NPC.

Co-infection with viruses and virus/virus direct or indirect interactions have been found in some malignancies, and a typical example for a malignant tumor due to virus/virus interactions is Kaposi's sarcoma-associated herpesvirus (KSHV)-induced Kaposi sarcoma in AIDS patients [30], [31]. Recent evidence indicates that individuals with HPV are at significantly higher risk of acquiring human immunodeficiency virus (HIV) infection [32], [33]. Co-infection of HPV and herpes simplex virus 1 (HSV-1) is also seen in a subset of HNC cases [5]. As HPV and EBV are the two most common viruses in HNC, it should be clarified whether co-infection leads to their interaction in HNC and why HPV prevalence has risen over the past few decades.

This prospective study investigated the frequency of EBV genotypes, *del-LMP-1* variants, and HPV genotypes in fresh-frozen samples from patients with HNC in order to analyze the effects of EBV and HPV co-infection on different types of HNC. The findings were then examined for associations with clinical features and prognosis.

## Materials and Methods

### Clinical samples and cell lines

We recruited 209 patients with HNC pathologically confirmed by the Department of Otorhinolaryngology, Head and Neck Surgery of the University of the Ryukyus, Japan, between December 2006 and March 2014. Written, informed consent was obtained from each patient and the research protocol was approved by the Ethics Committee of the University of the Ryukyus. Tissue samples obtained by biopsy or surgical excision were snap-frozen in liquid nitrogen and stored until further analysis.

The cell lines CaSki (ECACC, Salisbury, UK) and Raji (ATCC, Tokyo, Japan) were used as controls for the amplification of the HPV *L1* and *E6/E7* genes and the EBV *LMP-1* and *EBNA-3C* genes, respectively, and cultured according to the supplier's instructions.

### DNA extraction and PCR for detection of HPV DNA and EBV DNA

DNA was extracted from the samples and cells using the Gentra Purification Tissue Kit (Qiagen, Germantown, MD) according to the manufacturer's protocol. The presence and integrity of the DNA in all samples was verified by PCR â-globin gene amplification using primers PC04 and GH20 [34].

The presence of HPV DNA was analyzed by PCR using the general consensus primer sets *GP5+/GP6+* and *MY09/11* [35], [36]. DNA samples negative for HPV using *GP5+/GP6+* or *MY09/11* were re-amplified by (auto-) nested PCR using the *GP5+/GP6+* primer pair as previously described [37]. Water (negative control) and DNA from HPV-16-positive CaSki cells (positive control) were included in each amplification series. PCR products were purified and directly sequenced with an ABI PRISM 3130×l Genetic Analyzer (Applied Biosystems, Carlsbad, CA). Sequences were aligned and compared to those of known HPV types in the GenBank database using the BLAST program.

**Figure 1:**
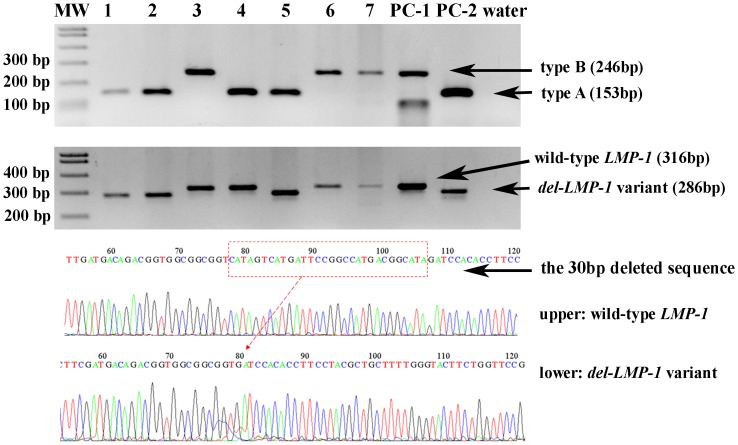
PCR results for EBV subtyping from the *EBNA-3C* gene (upper) and for the 30 bp deletion *LMP-1* variant (middle), and sequence analysis results for wild-type *LMP-1* and the 30 bp deletion *LMP-1* variant (lower Upper panel: the primers generated a specific 153 bp fragment from specimens containing type A EBV (samples 1, 2, 4, 5, and positive control PC-2) and a specific 246 bp fragment for specimens containing type B EBV (samples 3, 6, 7, and PC-1). Middle panel: a specific 316 bp band showed wild-type *LMP-1* (samples 3, 4, 6, 7, and positive control PC-1) and a specific 286 bp band indicated the 30 bp deletion *LMP-1* (samples 1, 2, 5, and PC-2). The products of positive controls were confirmed by direct sequencing. Lower panel: the 30 bp deletion sequence of *del-LMP-1* from wild-type LMP-1 by sequence analysis.

EBV DNA presence and typing was carried out by PCR using primers spanning the *EBNA-3C* gene as previous described [13]. Due to primer sites flanking regions of type-specific variation, the resulting PCR products were two different sizes derived from *EBV-A* (153 bp) and *EBV-B* (246 bp) (Figure 1). As positive controls, two EBV positive samples of type A and type B, confirmed by PCR and direct sequencing were included in each PCR reaction.

We also examined the *LMP-1* gene as previously reported [19]. The prototype Raji cell line (ATCC), which retains the carboxy terminal 30 bp, produces a 316 bp fragment and was used as a positive control for wild type *LMP-1*. Samples with a shorter PCR product length than that observed with Raji were believed to contain the *LMP-1* deletion (*del-LMP-1*, 286 bp fragment). In addition, a sample with a 30 bp deletion variant verified by PCR and direct sequencing was also included in each PCR reaction.

### Detection of HPV *E6/E7* mRNA by reverse transcription PCR

Total RNA extracted from tumor samples using the ToTALLY RNA™ kit (Ambion, Austin, TX) according to the manufacturer's protocols was suspended in 50 µL ultra-high quality diethyl pyrocarbonate-treated water.

Before cDNA synthesis, any residual DNA was removed by incubation with 1 U DNase I (Ambion) at room temperature for 25 min. cDNA was then synthesized from DNA-free total RNA using the RETROscript® Kit (Ambion) according to the manufacturer's instructions. To examine for the presence of contaminating DNA in RNA samples, all assays were performed both with and without reverse transcriptase.

To detect high-risk *E6/E7* mRNA transcripts, PCR was performed with cDNA obtained from HPV DNA-positive samples using the TaKaRa PCR Human Papillomavirus Typing Set (Takara Bio Inc., Otsu, Japan), which can identify high-risk HPV types 16, 18, 31, 33, 35, 52, and 58. To verify the HPV-16 *E6/E7* mRNA transcripts, the HPV-16 DNA-positive samples were also examined using a half-nested PCR approach with cDNA as previously described [38].

### ISH for *EBER*

The presence of EBV in cancer cells was confirmed by ISH for *EBER*. Briefly, 4 µm-thick sections from formalin-fixation paraffin-embedded blocks were deparaffinized, re-hydrated, and predigested with proteinase K. The hybridization solution containing fluorescein isothiocyanate (FITC)-conjugated EBV oligonucleotide probe (*EBER* PNA probe, cocktails of *EBER 1* and *2*; Dako, Glostrup, Denmark) was applied for 90 min at 55°C. A Dako ISH detection kit (code No. S5201) with rabbit F(ab′)2 anti-FITC antibody and enzyme substrate (5-bromo-4-chloro-3-indolylphosphate and nitroblue tetrazolium) was used to visualize the hybridization products. Stained slides were counterstained with Nuclear Fast Red (Vector Laboratories, Burlingame, CA). Dark-blue signals appearing within the nucleus were recognized as *EBER* positive.

### Statistical analysis

Descriptive statistics were used to characterize patient baseline characteristics. The Mann-Whitney U-test or Kruskal-Wallis test was used for continuous variables, and Pearson's Chi-square test or Fisher's exact test was used for categorical variables.

Recurrence-free survival was defined as the time from the end of treatment to cancer recurrence or last follow-up. Disease-specific survival was defined as the time from the end of treatment to subsidence of disease or last follow-up. Survival curves were evaluated by the Kaplan-Meier method, and survival distributions were compared using the log-rank test. *P* values less than 0.05 were considered significant. All statistical analyses were performed using SPSS software (SPSS for Windows version 12.0; SPSS, Inc., Chicago, IL).

## Results

### Clinicopathological characteristics of patients

Location of the primary tumor was the nasopharynx in 20 patients (9.6%), oropharynx in 74 (35.4%), hypopharynx in 50 (23.9%), larynx in 28 (13.4%), and oral cavity in 37 (17.7%). Of the 209 patients, 168 received curative treatment (concurrent chemoradiotherapy in 73, surgery and postoperative radiation or chemoradiation therapy (radiation dosage, 50–54 Gy) in 51, surgery alone in 27, and radiation therapy alone in 17), 30 patients received palliative treatment, and 11 had no treatment. Demographic and clinical characteristics are summarized in Table 1.

**Table 1:** Descriptive statistics of the main variables concerning patients and neoplasm parameters.

Characteristic	No. of patients (%) N = 209
Sex	
Male	177 (84.7)
Female	32 (15.3)
Age	
Median, years	65
Range, years	28–95
≤50	25
>50	184
Smoking^1^	
Never	40 (19.1)
≤400	32 (15.3)
>400	137 (65.6)
Alcohol use^2^	
Never	37 (17.7)
≤50	74 (35.4)
>50	98 (46.9)
Primary treatment	
Curative treatment	168 (80.4)
CCRT	73
Surgery with RT or CRT	51
Surgery	27
RT	17
Palliative treatment	30 (14.3)
No treatment	11 (5.3)
T classification	
T1	19 (9.1)
T2	78 (37.3)
T3	61 (29.2)
T4	51 (24.4)
Nodal status, n (%)	
N0 or N1	114 (54.5)
N2 or N3	95 (45.6)
TNM stage	
Early (I and II)	52 (24.9)
Advanced (III and IV)	157 (75.1)
Differentiation	
Well	93 (44.5)
Moderate	87 (41.6)
Poor	29 (13.9)
Tumor location	
Hypopharynx	50 (23.9)
Oropharynx	74 (35.4)
Oral cavity	37 (17.7)
Larynx	28 (13.4)
Nasopharynx	20 (9.6)
HPV^3^ DNA	
Negative	146 (69.9)
HPV-16	53 (25.3)
HPV-33	4 (1.9)
Other (e.g., HPV-35, HPV-56, HPV-58, and HPV-67)	6 (2.9)
EBV^4^ DNA	
Negative	63 (30.1)
Type A	107 (51.2)
Type B	27 (12.9)
Type A and B	12 (5.8)

1 Brinkman index: daily cigarettes × years.

2 Light drinker ≤50 g alcohol per day; heavy drinker>50 g alcohol per day.

3 HPV, human papillomavirus.

4 *EBNA*, Epstein-Barr nuclear antigen.

### HPV infection and genotypes in HNC

HPV DNA was detected in 30.1% (63/209) of the 209 patients, and the oropharynx was the site of highest prevalence (45.9%; 34/74). Genotyping of the neoplasms showed that most of the HPV DNA-positive specimens contained high-risk HPV-16 (86.9%; 53/63 tumors). The other HPV types included HPV-33 (4 cases), HPV-35 (2 cases), HPV-56 (1 case), HPV-58 (2 cases), and HPV-67 (1 case). No multiple infection or low-risk HPV types were found (Table 1).

Of the 63 HPV DNA-positive samples, 60 were available for RNA extraction and HPV *E6/E7* mRNA expression was found in 23 (38.3%) of them. Interestingly, most of the HPV *E6/E7* mRNA-positive tumors (87.0%; 20/23) derived from the oropharynx. While 60.6% (20/33) of HPV DNA-positive OPCs were positive for viral HPV mRNA expression, HPV *E6/E7* mRNA transcripts were found in only 3 of 27 (11.1%) non-oropharyngeal HNCs, deriving from the nasopharynx, larynx, or oral cavity (Table 2).

**Table 2:** Detection results of HPV DNA, mRNA, *EBNA*-3C, the 30 bp deletion LMP-1 variant, and *EBER* in different types of head and neck cancer.

Site of origin	HPV DNA, n (%)*	HPV mRNA, n (%)#	*EBNA-3C*, n (%)*	*del-LMP-1*, n (%)*	*EBER*, n (%)*	HPV DNA and *EBER*, n (%)*
Nasopharynx n = 20	6 (30.0%)	1 (16.7%)	19 (95%)	17 (85.0%)	12 (60.0%)	2 (10.0%)
Oropharynx n = 74	34 (45.9%)	20 (60.6%)	59 (79.7%)	47 (63.5%)	1 (1.4%)	0
Hypopharynx n = 50	8 (16.0%)	0	32 (64.0%)	27 (54.0%)	0	0
Larynx n = 28	4 (14.3%)	1 (33.3%)	14 (50.0%)	10 (35.7%	0	0
Oral cavity n = 37	11 (29.7%)	1 (9.1%)	22 (59.5%)	22 (59.5%)	0	0

HPV, human papillomavirus; *EBNA*, Epstein-Barr nuclear antigen; *LMP-1*, latent membrane protein-1; *EBER*, Epstein-Barr virus encoded RNA.

* Positive rate of the variables in different subsites of head and neck cancer.

# Positive rate of HPV mRNA in HPV DNA-positive patients; 3 cases with HPV DNA-positive derived from oropharynx, hypopharynx, and larynx were not sufficient to be examined for RNA extraction and *E6/E7* mRNA expression.

Analysis of the statistical correlation between HPV infection status and histoclinical features revealed no significant relationships between the presence of HPV DNA and sex, age, TNM stage, or nodal stage. HPV DNA was more likely to be found in nonsmokers or light smokers (i.e., Brinkman index <400; *P* = 0.030). In addition, significant correlations were observed between HPV status and tumor location (*P* = 0.002) and between HPV status and tumor differentiation (*P*<0.001).

### EBV infection, genotypes, and the 30 bp deletion *LMP-1* variant in HNC

As indicated in Table 2, PCR amplification of the *EBNA-3C* gene showed EBV DNA in 146 (69.9%) of the 209 HNC samples: 107 (73.3%) contained type A, 27 (18.5%) contained type B, and type A and type B were found together in 12 (8.2%) cases. We also investigated the prevalence of the *LMP-1* variant and found *del-LMP-1* in 124 (84.9%) of the 146 *EBNA*-positive cases. Almost all type A EBV cases (98.1%; 105/107) contained *del-LMP-1*, while just over half of type B cases (63.0%; 17/27) contained wild-type *LMP-1* (*P*<0.001).

The prevalence of EBV DNA by subsite was 95.0% (19/20), 79.7% (59/74), 64.0% (32/50), 50.0% (14/28), and 59.5% (22/37) in tumors from the nasopharynx, oropharynx, hypopharynx, larynx, and oral cavity, respectively. Of the 17 NPC specimens containing type A EBV, 16 (94.1%) showed *del-LMP-1*, including 6 WHO type I cases, 6 WHO type II cases, and 4 WHO type III cases.

**Figure 2:**
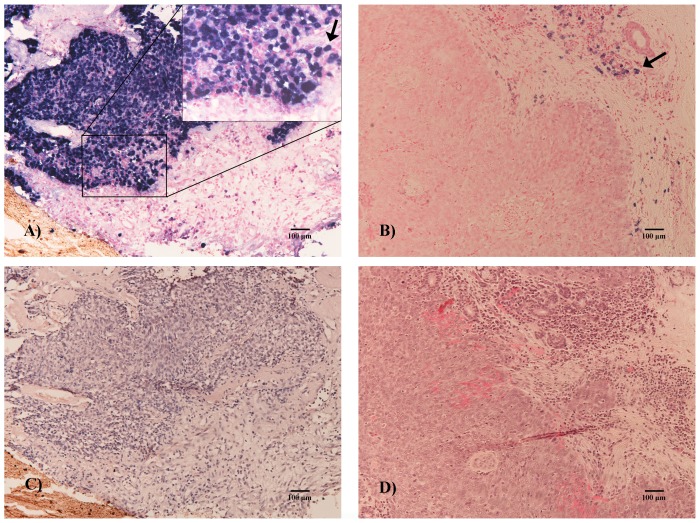
EBV encoded RNA in in situ hybridization. Micrograph A: most of the neoplasm cells were positive (nasopharyngeal carcinoma, ×100, bar = 100 µm), as indicated by the arrow at high magnification (×200). Micrograph B: although some positive lymphocytes are apparent (arrow), no neoplasm cells were positive. Micrographs C and D: Sections stained with hematoxylin and eosin (HE).

To determine the presence of EBV infection in tumor cells, *EBER* was also examined by ISH in all HNC samples (Figure 2). In contrast to the high prevalence of EBV DNA identified by PCR, only 6.2% (13/209) of HNCs were positive for *EBER*. EBV infection in HNC was mainly localized in the nasopharynx (92.3% [12/13] of *EBER*-positive tumors; nasopharynx vs non-nasopharynx HNC, *P*<0.001). *EBER* was detected in 60.0% (12/20) of patients with NPC, including 2 (22.2%, 2/9) with WHO type I, 6 (100%, 6/6) with WHO type II, and 4 (80%, 4/5) with WHO type III, but in only 1.4% (1/74) of patients with OPC. None of the *EBER*-positive cases derived from the hypopharynx, larynx, or oral cavity.

### HPV and EBV co-infection in HNC

Both HPV DNA and EBV DNA were detected by PCR in 45 (21.5%) of the total 209 patients. However, of the 13 *EBER*-positive HNCs, only 2 were HPV-16 positive and both derived from the nasopharynx. Since EBV infection in HNC is defined as *EBER*-positive tumor cells by ISH, the co-infection rates of HPV and EBV in HNC and NPC were only 1.0% and 10.0%, respectively (Table 3). Although HPV infection was most common in OPCs, none of the 34 HPV DNA-positive OPC cases was co-infected with EBV, as confirmed by *EBER* ISH.

**Table 3:** Co-infection of HPV and EBV in head and neck cancers, according to PCR for HPV DNA and ISH for *EBER*.

		*EBER* positive	*EBER* negative	*P value*
		n (%)		
HNC	HPV positive	2 (1.0)	61 (29.2)	0.352
	HPV negative	11 (5.2)	135 (64.6)	
NPC	HPV positive	2 (10.0)	4 (20.0)	0.161
	HPV negative	10 (50.0)	4 (20.0)	

HPV, human papillomavirus; EBV, Epstein-Barr virus; PCR, polymerase chain reaction; ISH, in situ hybridization; HNC, head and neck cancer; NPC, nasopharyngeal cancer; *EBER*, Epstein-Barr virus encoded RNA.

### Survival analysis

**Figure 3:**
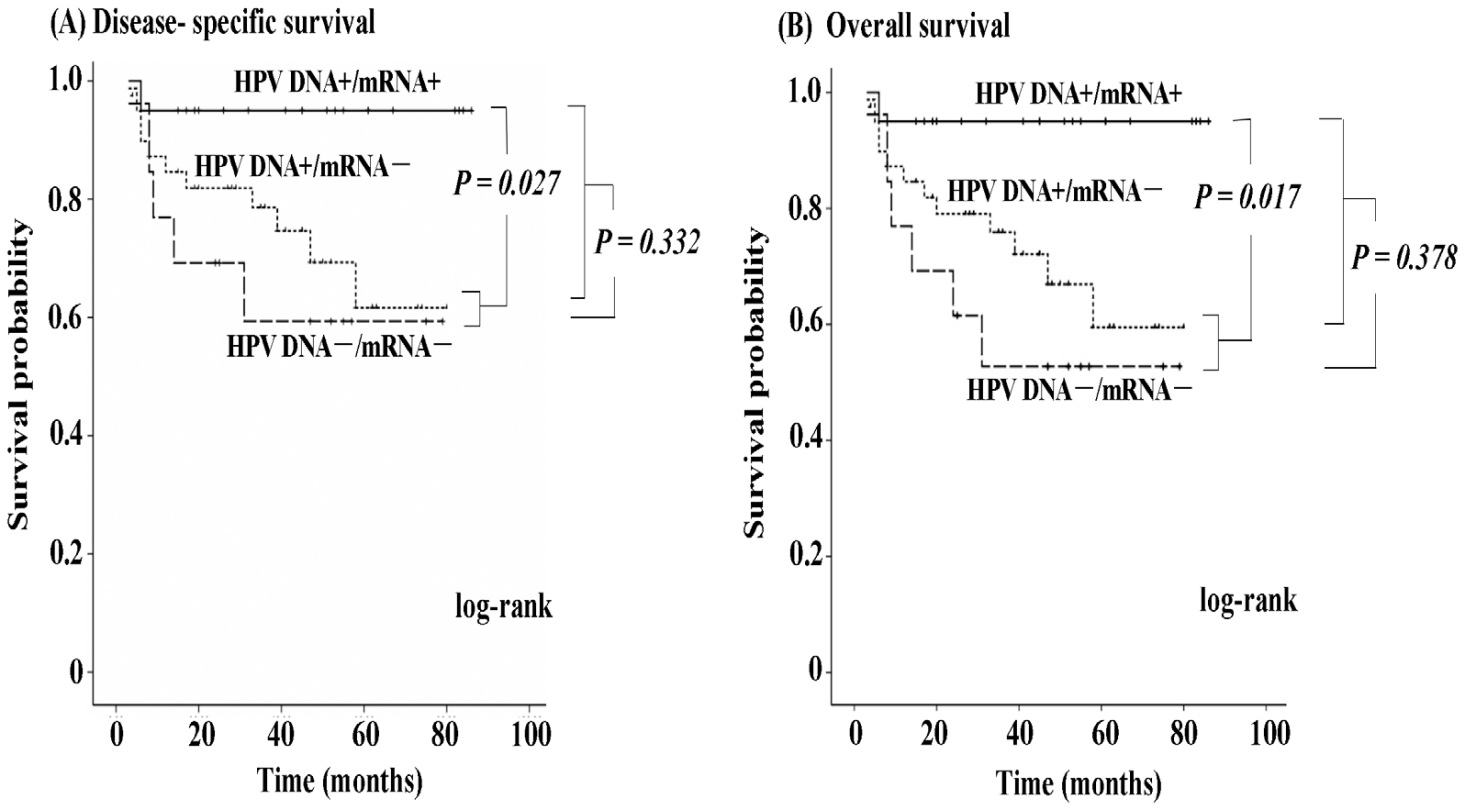
Kaplan-Meier curves of disease-specific survival (A) and overall survival (B) according to HPV status in all OPC patients. Disease-specific survival and overall survival rates were significantly better in HPV DNA-positive/mRNA-positive OPC patients than in HPV DNA-positive/mRNA-negative and HPV DNA-negative/mRNA-negative OPC patients. However, no significant differences in disease-specific survival or overall survival were found between HPV DNA-positive and HPV DNA-negative OPC patients.

Since there was insufficient tissue for mRNA detection in one case, we evaluated the treatment outcomes of 73 OPC patients based on HPV status. Fifty-four patients with previously untreated primary OPC had complete remission after receiving primary curative treatment, 5 showed persistent disease after curative treatment, 10 underwent palliative treatment, and 4 had no treatment. Median follow-up for patients whose data were censored was 41 months (range, 3–86 months). At latest follow-up, 18 (24.7%) of the 73 OPC patients had died of the disease and 2 had died of unrelated diseases. Kaplan-Meier analysis showed that no significant differences in disease-specific survival or overall survival between OPC patients with HPV DNA+ and OPC patients with HPV DNA- (*P* = 0.332 and *P* = 0.378, respectively; Fig. 3). However, compared with OPC patients with HPV mRNA− (i.e., both OPC patients with HPV DNA+/mRNA− and OPC patients with HPV DNA−/mRNA−), OPC patients with HPV DNA+/mRNA+ had better disease-specific and overall survival rates (*P* = 0.027 and *P* = 0.017, respectively). The 3-year disease-specific survival rate was 95.0% (95% CI = 85.5%–100%) in the cohort with HPV DNA+/mRNA+ and 73.8% (95% CI = 61.4%–86.1%) in the cohort with HPV DNA+/mRNA− or HPV DNA−/mRNA−.

To assess the independent predictive value of all these factors for disease-specific survival in OPC, univariate and multivariate analyses using Cox proportional-hazards models were performed. In univariate analysis, OPC patients with HPV DNA+/mRNA+ status demonstrated significantly higher disease-specific survival (*P* = 0.029; hazard ratio (HR) = 0.12; 95% confidence interval (CI) = 0.01–0.99) than the other OPC patients (Table 4). OPC patients categorized as stage T4, nodal stage 2–3, and heavy alcohol drinkers (alcohol per day>50 g) had significantly lower disease-specific survival (T4, *P* = 0.002, HR = 0.15, 95% CI = 0.04–0.50; nodal stage 2–3, *P* = 0.040, HR = 3.49, 95% CI = 1.01–12.03; heavy alcohol drinker, *P* = 0.040, HR = 3.49, 95% CI = 1.01–12.03) compared with patients categorized as stage T1–3, nodal stage 0–1, and light smokers and non-smokers. The final model of multivariate analysis using a Cox proportional hazards model for identification of fair disease-specific survival of OPCs showed that stage T1–3 (*P* = 0.002; adjusted HR = 0.22; 95% CI = 0.08–0.56) and HPV mRNA-positive status (*P* = 0.061; adjusted HR = 0.15; 95% CI = 0.02–1.10) predicted better disease-specific survival (Table 4).

**Table 4:** Univariate and multivariate analysis demonstrating the prognostic impact of HPV status on disease-specific survival in OPC.

	Univariate analysis (n = 73)*		Multivariate analysis (n = 73)	
Variable	*P*	HR (95% CI)	*P*	HR (95% CI)
HPV status (DNA+/mRNA+ vs. Others)	*0.029*	0.12 (0.01–0.99)	*0.061*	0.15 (0.02–1.10)
Others include DNA− and DNA+/mRNA−				
Age (>50 vs. ≤50 years)	*1.000*	1.07 (0.20–5.72)		
Sex (female vs. male)	*0.180*	2.51 (0.75–8.39)		
T stage (T1–T3 vs. T4)	*0.002*	0.15 (0.04–0.50)	*0.002*	0.22 (0.08–0.56)
Nodal stage (N2 or N3 vs. N0 or N1)	*0.040*	3.49 (1.01–12.03)	*0.125*	2.46 (0.78–7.76)
Smoking (heavy vs. light or never)	*0.059*	3.50 (0.90–13.56)		
Alcohol drinking (heavy vs. light or never)	*0.040*	3.49 (1.01–12.03)	*0.330*	1.78 (0.56–5.70)
Differentiation				
Well				
Moderate	*0.510*	1.82 (0.43–7.68)		
Poor	*0.422*	2.58 (0.51–13.01)		

HPV, human papillomavirus; HNC, head and neck cancer; HR, hazard ratio;

CI, confidence interval; OPC, oropharyngeal cancer.

*73/74 OPC patients were evaluated since one did not have sufficient tissue for mRNA detection.

**Figure 4:**
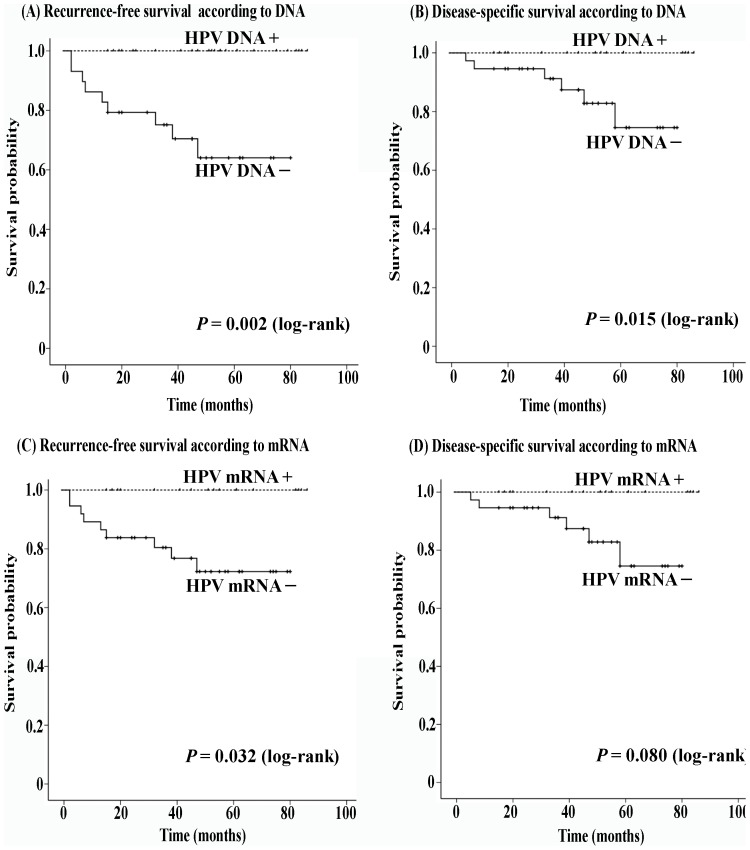
Kaplan-Meier curves of recurrence-free survival and disease-specific survival rates according to HPV status in OPC patients receiving curative treatment. After curative treatment, HPV DNA-positive OPC patients showed significantly better recurrence-free survival and disease-specific survival rates compared to HPV DNA-negative OPC patients. Similar results were found in HPV mRNA-positive OPC patients.

In addition, we assessed prognoses in the 54 OPC patients who had complete remission after receiving primary curative treatment. The 3-year recurrence-free survival was 100% for OPC patients with HPV DNA+ and 75.1% (95% CI = 59.1%–91.2%) for OPC patients with HPV DNA− (*P* = 0.002) (Figure 4A). Thus, the 3-year disease-specific survival in HPV DNA+ OPC patients was significantly better than in the HPV DNA- patients (100% vs 88.9%, *P* = 0.015) (Figure 4B). Interestingly, HPV mRNA+ OPC patients also exhibited significantly better recurrence-free survival than HPV mRNA- OPC patients (*P* = 0.032) (Figure 4C), and the 3-year recurrence-free survival rates were 100% and 80.4%, respectively. However, OPC patients with HPV mRNA+ demonstrated only a slightly stronger tendency toward fair disease-specific survival compared with OPC patients with HPV mRNA− (100% vs 91.2%, *P* = 0.080) (Figure 4D).

**Figure 5:**
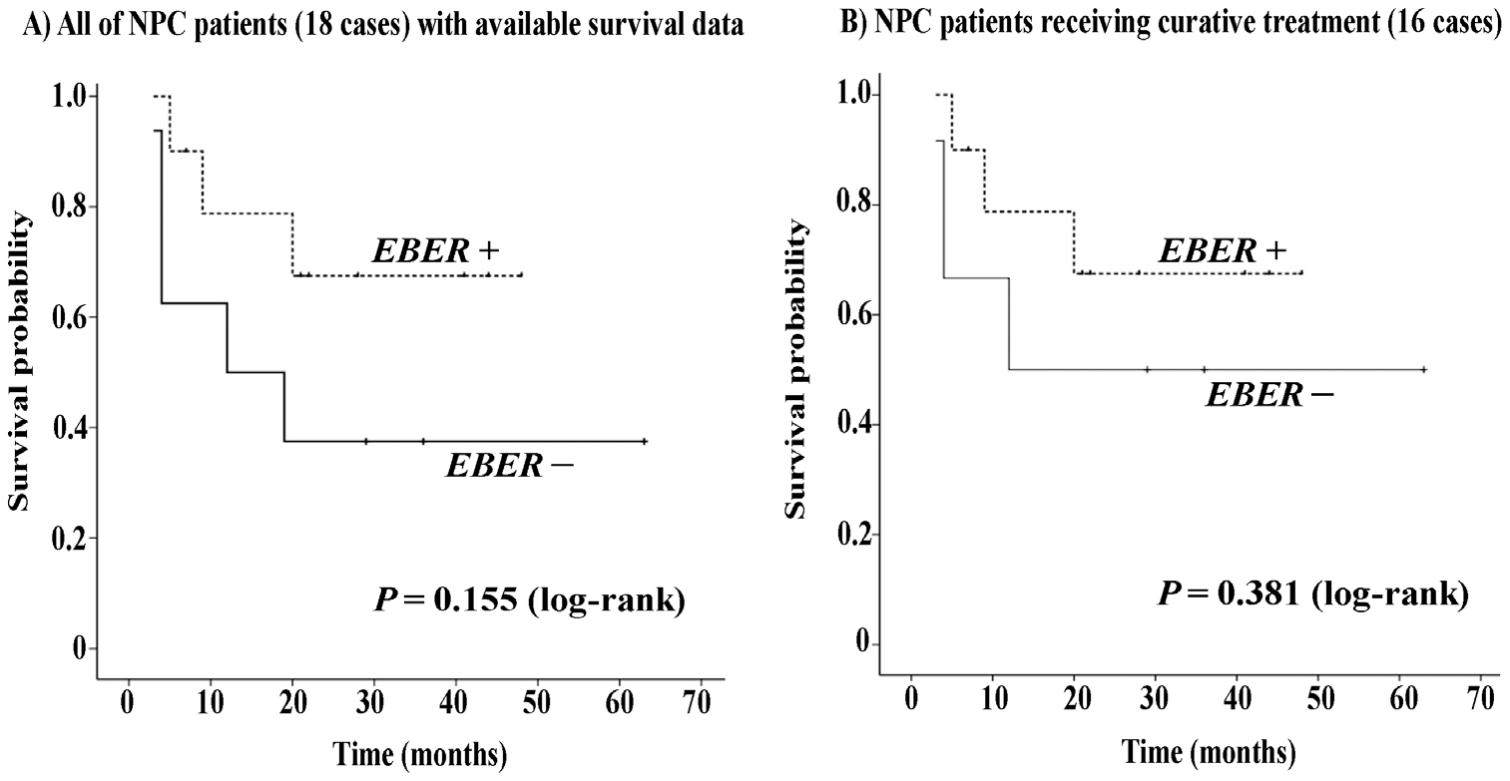
Kaplan-Meier curves of disease-specific survival according to *EBER* status in NPC patients. No significant differences in the rates of disease-specific survival were found between *EBER*-positive (tumor EBV infection) and *EBER*-negative NPC patients.

To assess the influence of EBV on survival for the patients with NPC, patients were stratified according to the detection of *EBER*. Among 20 patients with NPC, 18 were treated locally and treatment details and outcome were available from the time of diagnosis for these patients. The median follow-up time was 21 months (range, 3–63 months). Although patients with *EBER*-positive NPC showed a trend towards improved disease-specific survival compared with patients with *EBER*-negative NPC, no significant difference was found between these two cohorts (*P* = 0.155) (Figure 5A). Similarly, 16 cases received curative concurrent chemoradiotherapy for primary carcinoma, and no significant difference in disease-specific survival was found between the *EBER*-positive NPC and the *EBER*-negative NPC cohorts (*P* = 0.381) (Figure 5B).

## Discussion

The overall HPV prevalence in HNC was slightly higher in the present study (30.1%) than in international studies, which have reported 25.9% HPV-DNA-positive cases for these tumor entities [39]. Furthermore, our study findings were consistent with previous reports showing that the oropharynx was the subsite of the highest prevalence of HPV in HNC, and the most common viral genotype in HPV-associated HNC was HPV-16 [39], [40]. Unlike cervical cancer, where HPV *E6/E7* transcripts are identified in most HPV DNA-positive samples [41], only 38.4% of HPV DNA-positive HNC cases showed HPV *E6/E7* mRNA positivity in the present study. *E6/E7* mRNA is considered as a marker of HPV malignant transformation, and demonstrating oncogenic *E6/E7* transcription may suggest that HPV-driven carcinogensis is mechanistically possible, whereas HPV DNA-positive/RNA-negative results may simply reflect either low-level transcription or non-transcriptional active, and possibly a “passenger” infection. The fact that approximately 90% of cases with transcriptionally active HPV originated in the oropharynx supported the concept of a close relationship between HPV and OPC. Previous studies have demonstrated that the viral load of HPV DNA exhibits large variation among head and neck regions, and copy numbers of DNA in OPCs were much higher than in non-oropharynx HNCs. The relative low multiplicity of HPV infection in non-oropharynx head and neck sites, including the hypopharynx, larynx, and oral cavity, may correlate to low *E6/E7* mRNA expression in tumors at these sites [42], [43]. In addition, Wiest reported that most tumors that do not express *E6/E7* contain a mutated *p53* gene, in which the E6 gene is frequently disrupted. The lack of *E6/E7* expression might be due to the E6 gene disruption or alterations in transcriptional control [38].

In previous studies, patients with NPC in Taiwan commonly showed a higher prevalence of HPV (35–51%) [44], [45], [46] than those in Europe and the United States (9–27%) [47], [48], [49]. In addition, approximately 22% of oral cavity SCC cases in Taiwan harbor high-risk HPV DNA [50], [51]. Interestingly, in the present study, a relatively high incidence of HPV was also found in some non-oropharyngeal HNCs, such as NPC (30.0%) and oral cavity SCC (29.7%). Most of our patients were recruited from Okinawa, a southern island in Japan very near to Taiwan, which may suggest geographically and racially distinct HPV characteristics and that oncogenic HPV may be associated with a subset of carcinomas involving the nasopharynx and oral cavity. Given the low detection rates of *E6/E7* mRNA transcripts, however, the role of HPV in these non-oropharynx HNCs needs further clarification.

This study using PCR on fresh-frozen samples from NPCs showed that EBV type A is predominant and *del-LMP-1* is common in the Japanese population, findings which agree with data acquired from the same sort of specimens collected in Southeast Asia and North Africa [19], [20], [52], [53]. The opposite has been observed in samples from European EBV-associated NPC patients and healthy donors, where wild-type *LMP-1* isolates are more prominent than *del-LMP-1* variants [54]. The 30 bp deletion *LMP-1* variants exhibit transformation activity and induce tumorigenic changes, and the deletion protein has a longer half-life and a greater ability to activate NF-kB and JNK/AP1 in epithelial cells, thus contributing to a more malignant NPC phenotype [18], [55]. The distribution characteristics of EBV variants may suggest greater tumorigenic potential of *del-LMP-1* in endemic regions of Southeast Asia and North Africa compared with wild-type EBV. However, conflicting findings of no significant differences in the frequency of *del-LMP-1* in throat-wash samples between endemic and non-endemic regions in China [56] emphasize the need in future research to clarify whether *del-LMP-1* is indeed a NPC phenotype-correlated polymorphism and not a geographical- or ethnicity-related polymorphism.

Although just over half of cases with EBV type-B contained wild-type *LMP-1*, most of cases with EBV type-A contained *del-LMP-1*. Our findings are in concordance with a previous study from Korea [19], in which the majority (87%) of the type A EBV exhibited the *del-LMP-1* variant and the type B EBV exhibited wild-type *LMP-1* more frequently. Interestingly, wild-type *del-LMP-1* was not only found in 64.3% of type B EBV but was predominant (64.1%) in type A EBV in peripheral blood mononuclear cells from healthy carriers [57]. Moreover, Correa et al investigated 7 AIDS patients with primary central nervous system lymphoma and found that *del-LMP-1* was always detected with type B EBV [58], which was different from the findings in NPC. Taken together, the strong association of EBV type and *LMP-1* variants might be due to the intrinsic characteristics of EBV. The variation of *LMP-1*, together with polymorphisms in other regions of viral genome, may raise the possibility of virus strain-specific disease associations.

Our findings showed the same predominance of *del-LMP-1* in HNC types other than NPC. However, compared with the high detection rate (60%) of *EBER* in NPC, all non-nasopharynx HNCs but one were found to be *EBER* negative by ISH, which suggests that the 30 bp deleted *LMP-1* variants might be derived from non-malignant lymphocytes not cancer cells. Moreover, a high frequency of EBV and the *del-LMP-1* variant was observed in non-malignant tonsillar tissue in the present and another study [53]. The poor localization of EBV infection to a significant population of the tumor cells together with the negative ISH results present a strong argument against EBV playing a significant role in the pathogenesis of these non-nasopharynx HNCs. In addition, previous studies showed that EBV viral load in nasopharyngeal brushings in NPC patients was higher than in patients with non-NPC head and neck cancers [59]. The variation of multiplicity of infection may also contribute to the differences of *EBER* detection between NPC and non-NPC HNCs.

Although HPV and EBV co-infection has been investigated in a subset of HNC cases in previous studies, the results have been somewhat contradictory. Data from Taiwan and Morocco described such co-infection in NPC in up to 42% of the samples tested by PCR [45], [46], [60], while studies from Western countries including the United States, Denmark, and Greece demonstrated no co-infection with HPV and EBV in NPC [48], [49], [61]. These findings may be due to different sampling and preservation methods as well as the variety of detection methods used, including PCR, IHC and ISH [56]. To the best of our knowledge, this is the first work to identify co-infection with these two viruses by PCR and ISH—the gold standard methods for HPV and EBV, respectively—in various SCCs of the head and neck involving the nasopharynx, oropharynx, hypopharynx, or larynx. Our findings showed that only 10% of NPC cells and no SCCs at other sites harbored both HPV and EBV, findings which are in agreement with those of previous studies [62], [63], [64]. Apparently, a single PCR-based method may over-estimate the rate of HPV and EBV coexistence in HNC cells. In addition, the possibility of synergistic carcinogenic effects due to HPV and EBV in the head and neck is not supported because co-infection was not common in HNC.

It is well documented that tumors located in specific regions of the head and neck are more or less responsive to radiation and/or chemotherapeutic treatment [65], [66], and most HPV-positive and EBV-associated HNCs are found within the oropharynx and nasopharynx, respectively. We also assessed the roles of HPV status on the prognosis of OPC and EBV status on the prognosis of NPC. Regardless of treatment method and effect, disease-specific and overall survival outcomes were significantly better in patients with HPV mRNA+ than in those with HPV mRNA−. Positive HPV mRNA status also imparted significant benefits for recurrence-free survival in OPC patients who received curative treatment. Our findings suggest that HPV mRNA status (active infection) might be a more appropriate favorable prognostic indicator than HPV DNA of survival in OPC patients. Our study found no evidence of an association between EBV infection and NPC prognosis, which is in accordance with a previous report by Jeon et al. [19]. Interestingly, both studies showed a trend toward improved survival in the EBV infection cohort by Kaplan-Meier analysis, although Cox regression analysis did not support this prognostic value in Jeon et al.'s study. Given the small number of NPC patients in the present study, a greater number of cases and longer follow-up is needed in future studies examining the prognostic significance of EBV in NPC.

In conclusion, our findings indicate that although HPV and EBV are highly prevalent in cancers of the oropharynx and nasopharynx, respectively, HPV and EBV co-infection is rare in HNC, including at these two subsites. Oropharyngeal SCC with active HPV infection is associated with a highly favorable outcome, and an individualized therapeutic regimen based on HPV status and T stage in OPC patients should be evaluated and considered. While EBV type A and *del-LMP-1* are predominant in Japanese patients with NPC, EBV status was not prognostic in the NPC cohort.
