# Correction: Epstein-Barr Virus and Human Papillomavirus Infections and Genotype Distribution in Head and Neck Cancers

**DOI:** 10.1371/journal.pone.0118439

**Published:** 2015-03-05

**Authors:** The PLOS ONE Staff

In the Funding section, the grant number for the Science and Technology Development Center, Ministry of Education of the People’s Republic of China grant is missing. The correct grant number is: 20134433120021.
